# HIV treatment outcomes among people with initiation CD4 counts >500 cells/µL after implementation of *Treat All* in South African public clinics: a retrospective cohort study

**DOI:** 10.1002/jia2.25479

**Published:** 2020-04-21

**Authors:** Jienchi Dorward, Yukteshwar Sookrajh, Kelly Gate, Thokozani Khubone, Nomsa Mtshaka, Koleka Mlisana, Hope Ngobese, Nonhlanhla Yende‐Zuma, Nigel Garrett

**Affiliations:** ^1^ Centre for the AIDS Programme of Research in South Africa (CAPRISA) University of KwaZulu–Natal Durban South Africa; ^2^ Nuffield Department of Primary Care Health Sciences University of Oxford Oxford United Kingdom; ^3^ eThekwini Municipality Health Unit, eThekwini Municipality Durban South Africa; ^4^ Bethesda Hospital Ubombo South Africa; ^5^ Department of Family Medicine University of KwaZulu‐Natal Durban South Africa; ^6^ National Health Laboratory Service Johannesburg South Africa; ^7^ School of Laboratory Medicine and Medical Sciences University of KwaZulu‐Natal Durban South Africa; ^8^ Discipline of Public Health Medicine School of Nursing and Public Health University of KwaZulu‐Natal Durban South Africa

**Keywords:** HIV, Treat All, viral load, CD4, antiretroviral therapy, universal test and treat

## Abstract

**Introduction:**

The World Health Organisation recommends to *Treat All* people with HIV, irrespective of CD4 count. However, people with CD4 counts >500 cells/µL may be asymptomatic and therefore less motivated to adhere to antiretroviral therapy (ART). We aimed to assess whether people initiated with CD4 counts >500 cells/µL had worse treatment outcomes compared to those initiated at lower CD4 counts.

**Methods:**

We performed a retrospective cohort study among non‐pregnant adults initiating ART at eight public clinics in South Africa between September 2016, when *Treat All* was implemented, and August 2017. We assessed whether initiation CD4 count >500 cells/µL was associated with the outcomes of attrition (death, lost to follow‐up or treatment interruption >180 days), and viraemia >1000 copies/mL, by twelve months using Cox proportional hazards and Poisson regression models.

**Results and discussion:**

Among 4952 patients initiating ART, the median age was 32.4 years (interquartile range (IQR) 27.2 to 39.7), 58.9% were women and 30.3% had an initiation CD4 count >500 cells/µL. After twelve months, 3382 (68.3%) were retained in care, 303 (6.1%) had transferred to another clinic, 1010 (20.4%) were lost to follow‐up, 232 (4.7%) had a treatment interruption >180 days and 25 (0.5%) were known to have died. Overall, 1267 experienced attrition at a median time of 91 days (IQR 23 to 213), with 302 of these (23.8%) experiencing attrition immediately after their ART initiation visit. Among those in care at twelve months with viral load results, 4.6% had viraemia. In multivariable analysis, the hazard of attrition was similar between patients newly eligible for ART with CD4 counts >500 cells/µL compared to those with CD4 ≤500 cells/µL (adjusted hazard ratio 1.03, 95% confidence interval (CI) 0.90 to 1.17). The risk of viraemia was lower among patients with CD4 counts >500 cells/µL compared to those with CD4 ≤500 cells/µL (adjusted risk ratio 0.58, 95% CI 0.37 to 0.92).

**Conclusions:**

After implementation of *Treat All* in South African public clinics, we found that patients newly eligible for ART with initiation CD4 counts >500 cells/µL had comparable or better outcomes compared to those with lower CD4 counts. These finding support ongoing implementation of *Treat All* in our setting.

## 1 Introduction

In 2015 the World Health Organization (WHO) introduced its *Treat All* guidelines, which recommend antiretroviral therapy (ART) for all people living with HIV (PLHIV), including those with CD4 counts >500 cells/µL [1, 2]. Evidence for *Treat All* comes primarily from randomized controlled trials which demonstrated reductions in morbidity, mortality and onwards HIV transmission when patients were initiated on ART at higher CD4 counts [3, 4, 5].

*Treat All* has now been implemented in the majority of LMICs [2], but there is limited data regarding the impact on treatment outcomes in public sector ART programmes [6, 7]. In these settings, there has been concern that asymptomatic PLHIV with high CD4 counts could be less motivated to remain in care and adhere to treatment [8]. This could lead to viraemia and the development and spread of HIV drug resistance.

Therefore, we aimed to compare outcomes among PLHIV initiating ART at CD4 counts >500 cells/µL, against those with lower CD4 counts, after the implementation of *Treat All* in public sector settings.

## 2 Methods

### 2.1 Study design

We performed a retrospective cohort analysis using routinely collected data from eight public clinics in KwaZulu‐Natal, South Africa between 1 September 2016 and 30 June 2019. The Prince Cyril Zulu Clinic is a large urban clinic in central Durban, while Bethesda Hospital oversees seven primary care clinics in rural uMkhanyakude District, northern KwaZulu‐Natal. KwaZulu‐Natal has one of the highest HIV prevalences in the world at an estimated 27% among adults aged 15 to 49 [9]. All eight clinics provided HIV care according to South African National Department of Health guidelines, and implemented *Treat All* in September 2016 [10]. Prior to this, ART was restricted to people with CD4 counts ≤500 cells/µL, WHO Stage 3 or 4 disease, pregnant women, discordant couples or those with hepatitis B co‐infection [11].

### 2.2 Participants

We included all adolescents and adults >15 years old who were initiated on ART at the above clinics from 1 September 2016 to 31 August 2017, and followed them up for eighteen months. We excluded known pregnant women from the main analysis, as they have been eligible for ART irrespective of CD4 count since 2013 [12] and have different ART monitoring guidelines.

### 2.3 Data sources and data management

Demographic, clinical and laboratory data for all patients on ART in the South African public sector is routinely recorded at each clinical visit in a standardised clinical chart and subsequently into an electronic register (TIER.net) [13], from which we extracted data for this analysis. TIER.net data was validated monthly against clinic registers and chart reviews. Any missing laboratory results in TIER.net were searched for in the South African National Health Laboratory Service database. Anonymised data was analysed using Stata 14.0 (STATA Corp. College Park, TX, USA).

### 2.4 Variables

The main exposure variable was CD4 count at ART initiation. We included potential confounding variables recorded at ART initiation including age, sex, district, current active tuberculosis, ART regimen and time from first CD4 count to ART initiation, which we used as a proxy for time in pre‐ART care.

We used the outcome variable of attrition (a combination of death, lost to follow‐up, or treatment interruption), by twelve months post ART initiation. We defined attrition as 180 days without a visit, as this definition has been previously validated in a number of settings [14, 15, 16, 17]. Patients who subsequently restarted treatment after 180 days were defined as treatment interruptions. We analysed attrition as described by Grimsrud et al [16], using the date of the last visit as the date of attrition. To assess attrition at twelve months, we followed patients until eighteen months, to allow a window period to observe retention in care [16]. Patients whose records stated “transferred out” to another clinic were not defined as lost to follow‐up, but were censored on the day of last visit. We combined lost to follow‐up and death, as death is poorly recorded in the TIER.net register, and there is evidence that a significant proportion of patients lost to follow‐up in ART programmes have died [18].

For the outcome of viraemia, we used the WHO threshold of >1000 copies/mL. South African guidelines recommend viral load testing at six and twelve months after ART initiation, but in this programmatic setting, not all patients had viral loads taken exactly at these timepoints. Therefore, among patients who were retained in care, we included viral load results taken up to twelve months after ART initiation, and used the result closest to six months.

### 2.5 Statistical analysis

We used descriptive statistics to assess baseline demographic and clinical characteristics, and missing data. We used univariable and multivariable Cox proportional hazard models to test associations between the exposure of initiation CD4 count, and the outcome of time to attrition, adjusted for potential confounders. We selected variables for the univariable analysis and included them in the multivariable model based on data availability and clinical significance. Proportionality was assessed by fitting time‐dependent covariates in a model created by interacting baseline variables with survival time. Among those who were retained in care at twelve months and who had a viral load result, we employed a similar modelling approach, using univariable and multivariable Poisson regression models with robust standard errors, to analyse associations between initiation CD4 count and the outcome of viraemia >1000 copies/mL. We tested, *a priori*, for an interaction between time in pre‐ART care, and initiation CD4 count, and also performed sensitivity analyses using narrower CD4 count categories, and excluding two clinics with a high proportion of missing CD4 count data.

### 2.6 Ethical approval

Approval was granted by the Biomedical Research Ethics Committee of the University of KwaZulu‐Natal for use of routinely collected, anonymized data with a waiver for informed consent.

## 3 Results and discussion

### 3.1 Cohort characteristics

**Figure 1:**
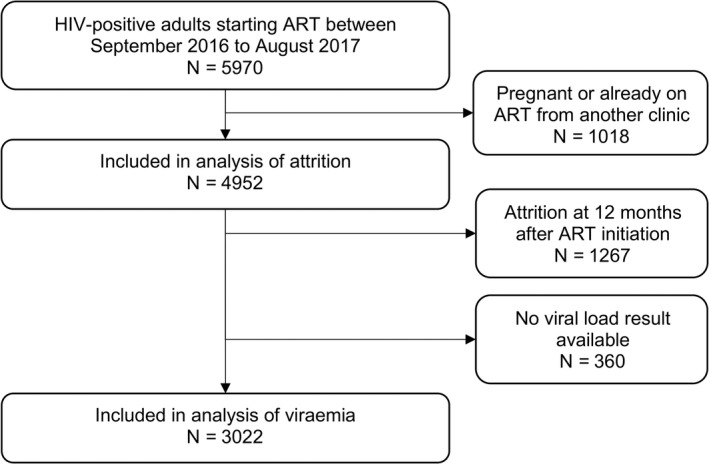
Flow diagram of numbers included in each analysis.

Between 1 September 2016 and 31 August 2017, 5970 adolescents and adults started ART at the eight clinics (Figure 1). Of these, we excluded 667 (11.2%) who had transferred in from another clinic, and a further 351 (6.6%) pregnant women. Of the remaining 4952 patients, 2968 (59.9%) were women and the median age was 32.4 years (IQR 27.2 to 39.7) (Table 1). Overall, 572 (11.2%) had tuberculosis at the time of ART initiation, and the majority (4855, 98.0%) received the standard first‐line regimen of tenofovir, emtricitabine and efavirenz. Among the 4552 (91.9%) who had a recorded initiation CD4 count, the median CD4 count was 366 cells/µL (IQR 195 to 570) and 32.9% had a CD4 count >500 cells/µL (Table 1). 480 patients (9.7%) were in pre‐ART care for more than six months before ART initiation. Of these, 291/480 (60.6%) had CD4 count >500 cells/µL, compared to 1208/4095 (29.5%) among those who were in pre‐ART care for less than six months. 3024 (61.1%) were initiated in the urban clinic and 1928 (38.9%) in the rural clinics, where there were slightly higher proportions of young adults aged 15 to 24 years, women, and patients with missing CD4 counts (Table 1), and a slightly lower proportion with tuberculosis.

**Table 1:** Baseline characteristics of study population, overall and stratified by district.

Variable	Overall (N = 4952) N (%)	Urban district (N = 3024, 61.1%) N (%)	Rural district (N = 1928, 38.9%) N (%)
Age			
≥45	656 (13.2)	389 (12.9)	267 (13.9)
35 to 44	1273 (25.7)	845 (28.0)	428 (22.2)
25 to 34	2216 (44.8)	1372 (45.4)	844 (43.8)
15 to 24	807 (16.3)	418 (13.8)	389 (20.2)
Sex			
Female	2968 (59.9)	1692 (56.0)	1276 (66.2)
Male	1984 (40.1)	1332 (44.0)	652 (33.8)
CD4, cells/µL			
≤500	3053 (61.7)	2036 (67.3)	1017 (52.8)
>500	1499 (30.3)	981 (32.4)	518 (26.9)
Missing	400 (8.1)	7 (0.2)	393 (20.4)
TB at ART initiation			
No	4401 (88.9)	2564 (84.8)	1837 (95.3)
Yes	551 (11.1)	460 (15.2)	91 (4.7)
Initiation >6 months after diagnosis			
No	4472 (90.3)	2771 (91.6)	1701 (88.2)
Yes	480 (9.7)	253 (8.4)	227 (11.8)
Initiated on tenofovir, emtricitabine & efavirenz			
Yes	4855 (98.0)	2939 (97.2)	1916 (99.4)
No	97 (2.0)	85 (2.8)	12 (0.6)

ART, antiretroviral therapy; TB, tuberculosis.

Most patients with missing initiation CD4 count came from two rural clinics, where 279/653 (42.7%) patients were missing CD4 data (Table S1). At the other six clinics, only 121/4299 (2.8%) were missing CD4 count. Between these two groups of clinics, the proportion with initiation CD4 count >500 cells/µL was similar (32.4% versus 33.0%, Table S1).

### 3.2 Attrition outcome

**Figure 2:**
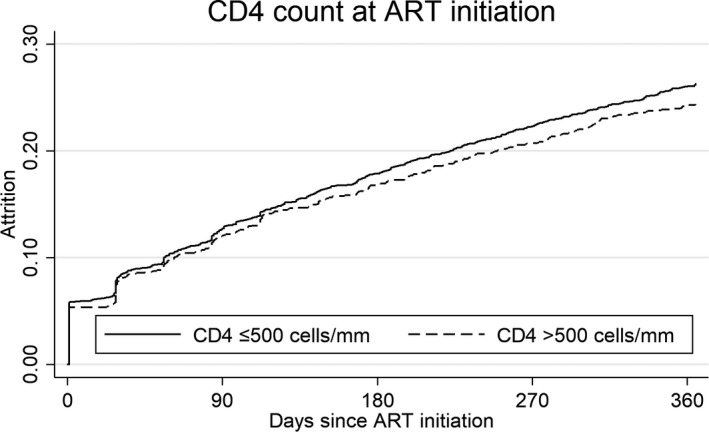
Kaplan‐Meier estimates of attrition by initiation CD4 count. Logrank *p*‐value 0.175. ART, antiretroviral therapy.

A total of 4952 patients were followed up to end‐point or censoring date over 3933 person‐years at risk, a mean of 290.1 days. At twelve months after initiation, 3382 (68.3%) were retained in care, 303 (6.1%) had transferred care to another clinic, 1010 (20.4%) were lost to follow‐up, 232 (4.7%) had a treatment interruption >180 days, and 25 (0.5%) were known to have died. Among the 1267 who experienced loss to follow‐up, treatment interruption or death, median time to attrition was 91 days (IQR 23 to 213), with 302 (23.8%) experiencing attrition immediately after their ART initiation visit. Of those who had a treatment interruption, the median duration was 224 days (IQR 196 to 288). In univariable analysis there was no association between initiation CD4 count and attrition (Table 2, Figure 2). Younger age and being male were associated with increased hazard of attrition, while longer time in pre‐ART care was associated with lower hazard of attrition. In multivariable analysis adjusting for age, sex, tuberculosis, district, time in pre‐ART care and ART regimen, the hazard of attrition was similar among patients with initiation CD4 count >500 cells/µL compared to those with CD4 ≤500 cells/µL (adjusted hazard ratio (aHR) 1.03, 95% CI 0.90 to 1.17, Table 2). There was higher attrition among patients with missing initiation CD4 counts (aHR 1.30, 95% CI 1.06 to 1.59), younger age (15 to 24 years vs. ≥45 years, aHR 1.83, 95% CI 1.48 to 2.27, 25 to 34 years vs. ≥45 years, aHR 1.41, 95% CI 1.17 to 1.70) and men (aHR 1.50, 95% CI 1.33 to 1.68) (Table 2). Longer time in pre‐ART care was associated with decreased hazard of attrition (aHR 0.67, 95% CI 0.54 to 0.84).

**Table 2:** Factors associated with attrition using Cox proportional hazard models (n = 4952)

Variable	Attrition at 12 months, n/N (%)	Unadjusted HR (95% CI)	*p*‐value	Adjusted HR (95% CI)^a^	*p*‐value
CD4, cells/µL					
≤500	777/3053 (25.5)	1	0.001	1	<0.001
>500	359/1499 (24.0)	0.92 (0.81 to 1.04)		1.03 (0.90 to 1.17)	
Missing	131/400 (32.8)	1.34 (1.12 to 1.62)		1.30 (1.06 to 1.59)	
Age, years					
≥45	135/656 (20.6)	1	<0.001	1	<0.001
35 to 44	287/1273 (22.6)	1.09 (0.89 to 1.34)		1.11 (0.91 to 1.37)	
25 to 34	589/2216 (26.6)	1.33 (1.11 to 1.61)		1.41 (1.17 to 1.70)	
15 to 24	256/807 (31.7)	1.65 (1.34 to 2.03)		1.83 (1.48 to 2.27)	
Sex					
Female	680/2968 (22.9)	1	<0.001	1	<0.001
Male	587/1984 (29.6)	1.37 (1.22 to 1.53)		1.50 (1.33 to 1.68)	
TB at ART initiation					
No	1128/4401 (25.6)	1	0.855	1	0.989
Yes	139/551 (25.2)	1.02 (0.85 to 1.21)		1.00 (0.83 to 1.20)	
District					
Urban	750/3024 (24.8)	1	0.088	1	0.336
Rural	517/1928 (26.8)	1.10 (0.98 to 1.23)		1.06 (0.94 to 1.20)	
Time to ART initiation					
<6 months	1180/4472 (26.4)	1	<0.001	1	<0.001
>6 months	87/480 (18.1)	0.63 (0.51 to 0.79)		0.67 (0.54 to 0.84)	
Initiated on tenofovir, emtricitabine & efavirenz					
Yes	1245/4855 (25.6)	1	0.543	1	0.594
No	22/97 (22.7)	0.88 (0.58 to 1.34)		0.89 (0.58 to 1.36)	

CI, confidence interval; HR, hazard ratio; TB, tuberculosis.

^a^ Adjusted for all other variables in the table.

In a sensitivity analysis using narrower CD4 categories, the hazard of attrition remained similar across CD4 count groups (compared to reference ≤200 cells/µL: 201 to 350 cells/µL, aHR 1.05, 95% CI 0.88 to 1.24; 351 to 500 cells/µL, aHR 0.92, 95% CI 0.77 to 1.10; >500 cells/µL aHR 1.01, 95% CI 0.86 to 1.20; n = 4952). In a second sensitivity analysis, there was some evidence that among people with longer time in pre‐ART care, there was a weak association between CD4 count >500 cells/µL and decreased attrition (aHR 0.68, 95% CI 0.43 to 1.07), compared to among people with shorter time in pre‐ART care (aHR 1.08, 95% CI 0.94 to 1.24, test for interaction *p* = 0.057, Table S2).

### 3.3 Viral load outcomes

Among the 3382 patients retained in care at twelve months, 3022 (89.4%) had a viral load at a median of 187 days (IQR 168 to 212) after ART initiation. The proportion with viral load results was similar between CD4 count groups (>500 cells/µL: 966/1063 (90.9%), ≤500 cells/µL: 1872/2078 (90.1%), *p* = 0.479). Of those with viral load results, 138 (4.6%) had viraemia >1000 copies/mL. In univariable analysis, the risk of viraemia was lower in patients with initiation CD4 count >500 cells/µL, and higher in patients with longer time in pre‐ART care, men, and those not on the standard first‐line regimen of tenofovir, emtricitabine and efavirenz (Table 3). In multivariable analysis, patients with initiation CD4 count >500 cells/µL had lower risk of viraemia compared to those with CD4 count ≤500 cells/µL (adjusted risk ratio (aRR) 0.58, 95% CI 0.37 to 0.92). Men (aRR 1.81, 95% CI 1.28 to 2.56) and patients not initiated on the standard first‐line ART regimen (aRR 2.54, 95% CI 1.24 to 5.19) had higher risk of viraemia (Table 3). In sensitivity analysis using narrower CD4 categories, the risk of viraemia was highest in patients with initiation CD4 counts ≤200 cells/µL (compared to reference ≤200 cells/µL: 201 to 350 cells/µL, aHR 0.44, 95% CI 0.27 to 0.71; 351 to 500 cells/µL, aHR 0.20, 95% CI 0.10 to 0.40; >500 cells/µL, aHR 0.32, 95% CI 0.19 to 0.52; N = 3022).

**Table 3:** Factors associated with viraemia using Poisson regression models with robust standard errors (n = 3022)

Variable	Viral load >1000 copies/mL n/N (%)	Unadjusted RR (95% CI)	*p*‐value	Adjusted RR (95% CI)^a^	*p*‐value
CD4, cells/µL					
≤500	100/1872 (5.3)	1	<0.001	1	0.011
>500	23/966 (2.4)	0.45 (0.29 to 0.70)		0.58 (0.37 to 0.92)	
Missing	15/184 (8.2)	1.53 (0.91 to 2.60)		1.60 (0.91 to 2.81)	
Age, years					
≥45	14/436 (3.2)	1	0.215	1	0.162
35 to 44	44/841 (5.2)	1.63 (0.90 to 2.94)		1.78 (0.99 to 3.19)	
25 to 34	66/1328 (5.0)	1.55 (0.88 to 2.72)		1.81 (1.03 to 3.18)	
15 to 24	14/417 (3.4)	1.05 (0.50 to 2.17)		1.37 (0.65 to 2.87)	
Sex					
Female	63/1898 (3.3)	1	<0.001	1	<0.001
Male	75/1124 (6.7)	2.01 (1.45 to 2.78)		1.81 (1.28 to 2.56)	
TB at ART initiation					
No	118/2698 (4.4)	1	0.142	1	0.469
Yes	20/324 (6.2)	1.41 (0.89 to 2.24)		1.19 (0.75 to 1.89)	
District					
Urban	86/1977 (4.4)	1	0.433	1	0.386
Rural	52/1045 (5.0)	1.14 (0.82 to 1.60)		1.18 (0.81 to 1.72)	
Time to ART initiation					
<6 months	131/2677 (4.9)	1	0.022	1	0.125
>6 months	7/345 (2.0)	0.41 (0.20 to 0.88)		0.55 (0.26 to 1.18)	
Initiated on tenofovir, emtricitabine & efavirenz					
Yes	131/2963 (4.4)	1	0.007	1	0.011
No	7/59 (11.9)	2.68 (1.31 to 5.49)		2.54 (1.24 to 5.19)	

CI, confidence interval; RR, risk ratio; TB, tuberculosis.

^a^ Adjusted for all other variables in the table.

### 3.4 Missing initiation CD4 counts

In sensitivity analyses which excluded two clinics containing 69.8% of all missing initiation CD4 counts, 97.2% of patients had initiation CD4 results and we found no meaningful differences in our results (Table S3 and S4).

## 4 Discussion

In this analysis of public sector clinics in a high HIV prevalence setting in South Africa, we found that patients initiated on ART with CD4 counts >500 cells/µL after *Treat All* implementation had similar attrition, and overall better viral load outcomes, compared to those with lower CD4 counts.

So far, evaluations of ART treatment outcomes among patients initiated with CD4 counts >500 cells/µL in LMICs have mainly been reported from well‐resourced research settings and large clinical trials of universal ART. For example, early ART initiation was acceptable in a South African research cohort [19], and adherence levels were high among patients initiated at CD4 counts >500 cells/µL in the Treatment as Prevention trial in KwaZulu‐Natal [20]. However, in a sub‐study of three South African clinics within the HPTN 071 (PopART) trial, patients initiated at CD4 counts >500 cells/µL had worse attrition [21], but comparable or better viral load outcomes compared to patients with lower CD4 counts [22]. The authors suggest that the novelty of providing ART for people with CD4 counts >500 cells/µL, before it became national policy, may have contributed to attrition in this group. In our study, in the early phase of *Treat All* implementation, patients initiated with high CD4 counts and in pre‐ART care for a longer time, had slightly lower attrition. We hypothesise that some of these patients remained engaged in pre‐ART care while waiting to become eligible for ART before *Treat All* implementation, and were therefore more likely to remain in care after they were initiated under *Treat All*.

Our findings are important as they provide reassurance that in programmatic settings people with high CD4 counts do not have significantly worse attrition, and have better viral suppression, which further supports earlier ART initiation. The inclusion of rural and urban public clinics, which provide care according to South African Department of Health guidelines, also supports the generalizability of our findings. Further strengths of our analysis include the large sample size, assessment of viral load outcomes and use of standard outcome definitions [16]. Our study has some limitations. First, we are likely to have overestimated loss to follow‐up as several studies have demonstrated high mortality and “silent transfers” to other clinics among people not retained in care in LMICs [18]. As we used routinely collected data from eight clinics, we were unable to search for silent transfers to other clinics, and did not have consent to search for deaths on the national registry. Second, we assessed early treatment outcomes, and further work will be required to assess if outcomes remain similar after longer follow‐up, and among people initiating ART with CD4 counts >500 cells/µL after the early phase of *Treat All* implementation. Third, initiation CD4 count was missing for some patients, and these patients had worse outcomes. This could have biased our findings, if patients with missing CD4 counts had a different CD4 count distribution. However, this is unlikely as missing data mainly occurred at two clinics, which had similar CD4 count distributions to the other six clinics. Staff may have placed less emphasis on CD4 count testing as it was no longer required to guide ART eligibility; a phenomenon reported in other programmatic studies of *Treat All* [6]. Additionally, in a South African study, the main reasons for missing CD4 testing appeared to be independent of CD4 count (e.g. patients not waiting, or clinic closing) [23].

Overall attrition at twelve months was slightly higher in our study (25.6%) compared to older studies in African settings (19.2%) [24] and occurred very early, although we found that 4.7% of patients did return to care after a treatment interruption. ART initiation on the same day as testing HIV positive may be associated with early attrition [25], and further work is needed to assess the impact of this policy, which was implemented in South Africa alongside *Treat All* [10]*.* However, among those retained in care in our study, viral suppression was high, which is similar to other LMIC settings [26]. Furthermore, we found higher risk of viraemia with non‐standard first‐line regimens, which may reflect differences in adherence due to side effects, or the presence of co‐morbidities that required use of other regimens. Lastly, while outcomes were similar in the rural and urban district, they were worse among younger people and men, which is consistent with other studies [27, 28, 29]. As implementation of *Treat All* intensifies, strategies to improve outcomes among these groups will be required.

## 5 Conclusions

In summary, we demonstrate that after implementation of *Treat All* in eight routine South African clinics, people starting ART with high CD4 counts had similar or better treatment outcomes compared to those with lower CD4 counts. Efforts to improve retention in care and implementation of *Treat All* should continue in order to achieve 90‐90‐90.

## Competing interest

We declare that we have no conflicts of interest.

## Authors’ contributions

JD, YS, KG and NG conceptualized the study. TK, NM, YS, KG, KM and HN oversaw data collection. TK, NM, KM and HN oversaw curation of the data. JD and NYZ analysed the data. JD drafted the manuscript. All authors critically reviewed and edited the manuscript.

## Abbreviations

aHR, Adjusted hazard ratio; aRR, Adjusted risk ratio; ART, Antiretroviral therapy; CI, Confidence interval; HIV, Human Immunodeficiency Virus; IQR, Interquartile range; LMICs, Low‐ and middle‐income countries; PLHIV, People living with HIV; WHO, World Health Organization.

## Supporting information


**Table S1.** Distribution of known CD4 counts at clinics with near complete CD4 count data compared to clinics with high levels of missing data
**Table S2.** Attrition and viraemia by CD4 count, among patients initiated within six months of HIV diagnosis and patients initiated over six months after diagnosis
**Table S3.** Sensitivity analysis of factors associated with attrition at six public clinics with near complete initiation CD4 count data (n = 4299)
**Table S4.** Sensitivity analysis of factors associated with viraemia at six public clinics with near complete initiation CD4 count data (n = 2678)
