# Supplementary material for: HIV treatment outcomes among people with initiation CD4 counts >500 cells/µL after implementation of Treat All in South African public clinics: a retrospective cohort study
Source: J Int AIDS Soc. 2020 Apr 21;23(4):e25479. doi: 10.1002/jia2.25479 (PMC7174836; doi:10.1002/jia2.25479)
Supplement: Supplementary file 1 — Table S1. Distribution of known CD4 counts at clinics with near complete CD4 count data compared to clinics with high levels of missing data Table S2. Attrition and viraemia by CD4 count, among patients initiated within six months of HIV diagnosis and patients initiated over six months after diagnosis Table S3. Sensitivity analysis of factors associated with attrition at six public clinics with near complete initiation CD4 count data (n = 4299) Table S4. Sensitivity analysis of factors associated with viraemia at six public clinics with near complete initiation CD4 count data (n = 2678) [file JIA2-23-e25479-s001.docx]

# Supplementary Tables

*Table S1: Distribution of known CD4 counts at clinics with near complete CD4 count data compared to clinics with high levels of missing data*

|  | | Health facility | |
| --- | --- | --- | --- |
|  | | Six clinics with near complete CD4 data  (4178/4299, 97.2% complete) | Two clinics with low levels of complete CD4 data  (374/653, 57.3% complete) |
| CD4, cells/µL | ≤500 | 2800 (67.0) | 253 (67.6) |
|  | >500 | 1378 (33.0) | 121 (32.4) |
| Total |  | 4178 (100) | 374 (100) |

*Table S2: Attrition and viraemia by CD4 count, among patients initiated within six months of HIV diagnosis and patients initiated over six months after diagnosis.*

|  | | Attrition amongst patients initiated within six months of diagnosis | | Attrition amongst patients initiated over six months after diagnosis | |
| --- | --- | --- | --- | --- | --- |
|  |  | n/N (%) | Adjusted HR*^†^ (95% CI) | n/N (%) | Adjusted HR*^†^ (95% CI) |
| CD4^†^, cells /µL n=4552 | <500 | 742/2887 (25.7) | 1 | 35/166 (21.1) | 1 |
|  | >500 | 319/1208 (26.4) | 1.08 (0.94-1.24) | 40/291 (13.8) | 0.68 (0.43-1.07) |
|  | | Viraemia amongst patients initiated within six months of diagnosis | | Viraemia amongst patients initiated over six months after diagnosis | |
|  |  | n/N (%) | Adjusted RR*^‡^ (95% CI) | n/N (%) | Adjusted RR*^‡^ (95% CI) |
| CD4^‡^, cells /µL n=2838 | <500 | 97/1761 (5.5) | 1 | 3/111 (2.7) | 1 |
|  | >500 | 20/743 (2.7) | 0.59 (0.37-0.94) | 3/223 (1.4) | 0.56 (0.12-2.72) |

*adjusted for age, sex, TB at ART initiation, district, and ART regimen; ^†^p-value for interaction 0.057; ^‡^p value for interaction 0.956. HR hazard ratio; CI confidence interval; RR risk ratio

*Table S3: Sensitivity analysis of factors associated with attrition at six public clinics with near complete initiation CD4 count data (n=4299)*

| **Variable** | | **Attrition at 12 months, n/N (%)** | **Unadjusted HR**  **(95% CI)** | **p-value** | **Adjusted HR**  **^†^ (95% CI)** | **p-value** |
| --- | --- | --- | --- | --- | --- | --- |
| CD4, cells/µL | <500 | 720/2800 (25.7) | 1 | <0.001 | 1 | 0.005 |
|  | >500 | 326/1378 (23.7) | 0.89 (0.78-1.02) |  | 1.01 (0.88-1.16) |  |
|  | Missing | 49/121 (40.5) | 1.78 (1.32-2.37) |  | 1.72 (1.27-2.33) |  |
| Age, years | ≥45 | 114/570 (20.0) | 1 | <0.001 | 1 | <0.001 |
|  | 35-44 | 256/1125 (22.8) | 1.13 (0.91-1.41) |  | 1.14 (0.92-1.43) |  |
|  | 25-34 | 515/1936 (26.6) | 1.37 (1.12-1.68) |  | 1.44 (1.17-1.76) |  |
|  | 15-24 | 210/668 (31.4) | 1.67 (1.33-2.10) |  | 1.87 (1.48-2.35) |  |
| Sex | Female | 568/2532 (22.4) | 1 | <0.001 | 1 | <0.001 |
|  | Male | 527/1767 (29.8) | 1.42 (1.26-1.59) |  | 1.54 (1.36-1.74) |  |
| TB at ART initiation | No | 960/3765 (25.5) | 1 | 0.821 | 1 | 0.875 |
|  | Yes | 135/534 (25.3) | 1.02 (0.85-1.22) |  | 0.99 (0.82-1.19) |  |
| District | Urban | 750/3204 (24.8) | 1 | 0.081 | 1 | 0.196 |
|  | Rural | 345/1275 (27.1) | 1.12 (0.99-1.27) |  | 1.09 (0.96-1.25) |  |
| ART initiation >6 months from HIV diagnosis | No | 1020/3871 (26.4) | 1 | <0.001 | 1 | <0.001 |
|  | Yes | 75/428 (17.5) | 0.61 (0.48-0.77) |  | 0.65 (0.51-0.82) |  |
| Initiated on tenofovir, emtricitabine & efavirenz | Yes | 1073/4204 (25.5) | 1 | 0.621 | 1 | 0.636 |
|  | No | 22/95 (23.2) | 0.90 (0.59-1.37) |  | 0.90 (0.59-1.38) |  |

^†^Adjusted for all other variables in the table.

HR Hazard Ratio; CI confidence interval; TB Tuberculosis

*Table S4: Sensitivity analysis of factors associated with viraemia at six public clinics with near complete initiation CD4 count data (n=2678)*

| **Variable** | | | **Viral load >1000 copies/ml n/N (%)** | | **Unadjusted RR**  **(95% CI)** | **p-value** | **Adjusted RR* (95% CI)** | **p-value** |
| --- | --- | --- | --- | --- | --- | --- | --- | --- |
| CD4, cells/µL | ≤500 | | 90/1723 (5.2) | | 1 | <0.001 | 1 | 0.010 |
|  | >500 | | 22/903 (2.4) | | 0.47 (0.29-0.74) |  | 0.61 (0.38-0.97) |  |
|  | Missing | | 6/52 (11.5) | | 2.21 (1.01-4.81) |  | 2.35 (1.04-5.28) |  |
| Age, years | ≥45 | | 12/386 (3.1) | | 1 | 0.222 | 1 | 0.171 |
|  | 35-44 | | 35/754 (4.6) | | 1.49 (0.78-2.84) |  | 1.62 (0.85-3.09) |  |
|  | 25-34 | | 60/1178 (5.1) | | 1.64 (0.89-3.01) |  | 1.88 (1.02-3.47) |  |
|  | 15-24 | | 11/360 (3.1) | | 0.98 (0.44-2.20) |  | 1.25 (0.55-2.84) |  |
| Sex | Female | | 57/1664 (3.4) | | 1 | 0.002 | 1 | 0.022 |
|  | Male | | 61/1014 (6.0) | | 1.76 (1.23-2.50) |  | 1.54 (1.06-2.24) |  |
| TB at ART initiation | No | | 99/2362 (4.2) | | 1 | 0.138 | 1 | 0.510 |
|  | Yes | | 19/316 (6.0) | | 1.43 (0.89-2.31) |  | 1.17 (0.73-1.90) |  |
| District | | Urban | | 86/1977 (4.4) | 1 | 0.812 | 1 | 0.593 |
|  |  | Rural | | 32/701 (4.6) | 1.05 (0.71-1.56) |  | 1.12 (0.74-1.71) |  |
| ART initiation >6 months from HIV diagnosis | | No | | 113/2366 (4.8) | 1 | 0.016 | 1 | 0.086 |
|  |  | Yes | | 5/312 (1.6) | 0.34 (0.14-0.82) |  | 0.45 (0.18-1.12) |  |
| Initiated on tenofovir, emtricitabine & efavirenz | | Yes | | 111/2619 (4.2) | 1 | 0.005 | 1 | 0.011 |
|  |  | No | | 7/59 (11.9) | 2.80 (1.36-5.75) |  | 2.56 (1.24-5.28) |  |

*Adjusted for all other variables in the table.

RR Risk Ratio; CI confidence interval; TB Tuberculosis
